# Poly(hydroxybutyrate-*co*-hydroxyvalerate) Porous Matrices from Thermally Induced Phase Separation

**DOI:** 10.3390/polym12122787

**Published:** 2020-11-25

**Authors:** Reza Zeinali, Mohammad Taghi Khorasani, Aliasghar Behnamghader, Mohammad Atai, Luis del Valle, Jordi Puiggalí

**Affiliations:** 1 Department of Biomedical Engineering, Science and Research Branch, Islamic Azad University, Tehran 1477893855, Iran; 2 Departament d’Enginyeria Química, Universitat Politècnica de Catalunya, Escola d’Enginyeria de Barcelona Est-EEBE, 08019 Barcelona, Spain; luis.javier.del.valle@upc.edu; 3 Department of Biomaterials, Iran Polymer and Petrochemical Institute, Tehran 1497713115, Iran; m.khorasani@ippi.ac.ir; 4 Research Department of Nanotechnology and Advanced Materials, Materials and Energy Research Center, Karaj 3177983634, Iran; a-behnamghader@merc.ac.ir; 5 Department of Polymer Science, Iran Polymer and Petrochemical Institute, Tehran 1497713115, Iran; m.atai@ippi.ac.ir

**Keywords:** polyhydroxyalkanoates, poly(hydroxybutyrate-*co*-hydroxyvalerate), thermally induced phase separation, freeze drying, scaffolds, cooling rate, pore morphology

## Abstract

Thermally induced phase separation followed by freeze drying has been used to prepare biodegradable and biocompatible scaffolds with interconnected 3D microporous structures from poly(hydroxybutyrate-*co*-hydroxyvalerate) (PHBV) copolymers containing 5 and 12 wt % of 3-hydroxyvalerate (HV). Solutions of PHBV in 1,4-dioxane, underwent phase separation by cooling under two different thermal gradients (at −25 °C and −5 °C). The cloud point and crystallization temperature of the polymer solutions were determined by turbidimetry and differential scanning calorimetry, respectively. Parameters affecting the phase separation mechanism such as variation of both the cooling process and the composition of the PHBV copolymer were investigated. Afterwards, the influence of these variables on the morphology of the porous structure and the final mechanical properties (i.e., rigidity and damping) was evaluated via scanning electron microscopy and dynamic mechanical thermal analysis, respectively. While the morphology of the scaffolds was considerably affected by polymer crystallization upon a slow cooling rate, the effect of solvent crystallization was more evident at either high hydroxyvalerate content (i.e., 12 wt % of HV) or high cooling rate. The decrease in the HV content gave rise to scaffolds with greater stiffness because of their higher degree of crystallinity, being also noticeable the greater consistency of the structure attained when the cooling rate was higher. Scaffolds were fully biocompatible supports for cell adhesion and proliferation in 3D cultures and show potential application as a tool for tissue regeneration.

## 1. Introduction

Numerous applications of natural-origin polymers in the biomedical field (e.g., drug and cell carriers) are focusing the attention of researchers [1]. Polyhydroxyalkanoates (PHAs) are a large sub-branch of natural polyesters that can be extracted from bacteria or genetically modified plants. The poly(3-hydroxybutyrate) (PHB) homopolymer and the poly(3-hydroxybutyrate-*co*-3- hydroxyvalerate) (PHBV) copolymer are the members of the PHAs family with the highest applications. The main problem of PHB concerns to its high brittleness and crystallinity and therefore copolymers incorporating small percentages of 3-hydroxyvalerate (HV) units (i.e., PHBV copolymers) are being commercialized since they can also be easily produced by bacteria (e.g., *Escherichia coli*, *Paracoccus denitrificans*, *Ralstonia eutropha*) as storage products.

Due to properties such as biocompatibility, biodegradability, non-toxicity, and piezoelectricity, the use of PHBV copolymers in a variety of medical fields including surgical sutures, wound dressings, controlled release, and tissue engineering has been reported [2,3,4,5,6]. Chemical and mechanical properties of PHBV copolymers can logically be controlled in function of the HV content. Despite relatively high crystallinity levels can be achieved at various HV ratios, it is obvious that the increase of the comonomer HV content lead to polymers with lower degrees of crystallinity and melting temperatures. In addition, besides exhibiting full degradability in aqueous environments and producing non-toxic by-products, the degradation rate of copolymers can be tuned by varying the HV content [7,8,9].

The thermally induced phase separation (TIPS) technique has extensively been used in non-biomedical fields for fabricating synthetic membranes. Applications in the biomedical sector are also habitual as for example for the development of drug delivery systems. Specifically, the methodology has been employed to prepare microspheres incorporating pharmaceutical and biological agents [10,11]. Today, TIPS is a common technique to fabricate porous scaffolds for tissue engineering applications [12,13,14,15]. This method is based upon thermodynamic demixing of a homogeneous polymer solution into polymer-rich and polymer-lean (solvent-rich) phases [16]. The solvent in the polymer-lean phase can subsequently be eliminated by extraction, evaporation, or sublimation [17], leaving behind a highly porous polymer network [18,19]. TIPS experimentally allows controlling the final structure of the scaffold in terms of morphology, average pore size and degree of interconnection [20]. The final structure and pore morphology of the phase-separated polymer matrices are greatly dependent on the combination of the selected polymer and solvent system, the polymer concentration, the phase-separation temperature and the temperature gradient applied to the polymer solution [21].

Various biodegradable polymers have been considered to fabricate three-dimensional scaffolds through the TIPS technique and investigated for tissue regeneration applications [22]. In this regard, the application of the phase separation method for scaffolding purposes has been reported for several biodegradable polyesters, especially polylactide (PLA) and poly(lactide-*co*-glycolide) (PLGA). Depending on the polymer system and phase separation conditions, these 3D polyester scaffolds can structurally be classified as solid-walled isotropic and anisotropic (like microtubular), fibrous, nanofibrous, and platelet-like architectures [23,24,25,26,27]. Moreover, the different types of TIPS techniques—i.e., solid–liquid [23,24,25], liquid–liquid [26,27], and crystallization-induced phase separations [27]—have been used for creating different micro- and nano-structured polymer constructs. Organic solvents with high freezing points like 1,4 dioxane or benzene and others with low freezing points like THF, DMF, and pyridine have successfully been used to fabricate scaffolds by solid–liquid and liquid–liquid phase separations, respectively [23,24,25,26,27].

The phase separation procedure has also been applied to different scaffolding materials based on polyhydroxyalkanoates. Thus, the fabrication of nanofibrous and microtubular architectures have mainly been reported for systems based on PHB, poly(3-hydroxybutyrate-*co*-3-hydroxyhexanoate) (PHBHx) and poly(3-hydroxybutyrate-*co*-4-hydroxybutyrate) (P(3HB-4HB)) [28,29]. Nevertheless, the fabrication of phase-separated porous scaffolds made of PHBV copolymers, has received less attention. Furthermore, scarce studies can be found evaluating TIPS-obtained PHBV scaffolds in terms of pore morphology and paying attention to the copolymer properties and the phase separation conditions.

In the present study, the potential of PHBV copolymers and TIPS technique to develop interconnected 3D networks with solid-wall and platelet-like structures has been appraised. We have specifically addressed how altering the quenching temperatures and copolymer characteristics have a considerable effect on the phase separation process and the scaffold properties. The disparities observed in the morphological features and mechanical properties of resulting scaffolds were discussed with respect to thermodynamic and kinetic conditions of phase separation.

1,4-dioxane is used as a solvent for a variety of practical applications and can be found for example at minimum levels in cosmetics and personal care products. Some toxicologic effects of dioxane have been recognized, being consequently a potential health concern that received the attention of FDA. Despite no specific law requirements have been formulated, manufacturers have been encouraged to remove dioxane from technological processes [30]. Therefore, we have paid also special attention, through biocompatibility tests, to ensure a complete solvent removal in the final processed scaffolds.

## 2. Materials and Methods

### 2.1. Materials and Scaffold Preparation

Poly(3-hydroxybutyrate-*co*-3-hydroxyvalerate)s containing 5 and 12 molar percentages of 3-hydroxyvalerate were purchased from Sigma-Aldrich (St. Louis, MO, USA) and were used for fabrication of polymeric scaffolds. The molecular weights of PHBV(5%HV) and PHBV(12%HV) were 320 kDa and 240 kDa and their corresponding melting points were 165 °C and 161 °C, respectively. 1,4-dioxane was used as solvent and was supplied by Acros Organics (Morris Plains, NJ, USA), with linear formula of C_4_H_8_O_2_, molecular weight of 88.11 g/mol, density of 1.033 g/mL, melting point of 12 °C, and purity of 99.5%. The polymers and the solvent were used without further purification.

For preparing polymeric foams, the corresponding polymer solutions were cooled until phase separation occurred. Subsequently, porous structures were achieved after removing the solvent. Specifically, both PHBV(5%HV) and PHBV(12%HV) copolymers were dissolved at a concentration of 2% (*w*/*v*) in 1,4-dioxane by heating and stirring. When the temperature reached about 70 °C, a clear homogenous polymer solution was attained. The solutions (0.4 mL) were poured into a cylinder-shaped glass container with a diameter of 14 mm and height of 40 mm and then sealed. The samples first were cooled spontaneously to the room temperature and then immediately incorporated to the corresponding cooling devices preset on −5 °C or −25 °C. In this process, phase separation occurred during cooling. Note that this process occurred under two different thermal gradients, that is, from room temperature to −5 °C and −25 °C. Afterwards, samples were kept at rest for 24 h at the selected final temperature. Finally, the samples were lyophilized (Gamma 2-16 LSC, Martin Christ, Osterode am Harz, Germany) for 40 h. The resulting porous scaffolds were dried in a vacuum oven at room temperature to reach a constant weight.

### 2.2. Cloud Point and Cooling Rate Determination and DSC Analysis of Polymer Solutions

The cloud point of polymer solutions was evaluated by visual turbidimetry. In order to predict the location of binodal curve at relatively low concentrations of the PHBV-dioxane phase diagram, the cloud points of 1–10% (*w*/*v*) solutions were determined. The solutions were poured into transparent sealed glass containers and then transferred to a refrigerated incubator to reach equilibrium conditions through a controllable slow cooling. The incubator was preset at 35 °C and programmed to be cooled at a rate of 0.033 °C/min (i.e., 1 °C each 30 min). The temperature at which the clear solution became turbid was recognized as the cloud point. At least three independent turbidimetric assays per sample were performed, being the results averaged and the standard deviations obtained.

Cooling rate of the different polymer solutions was determined using a digital thermometer (ESCORT 20 T/C, EIC, Taipei, Taiwan) inserted into the center of the tube containing the respective solutions. The thermometer was connected to the computer and plotted the cooling diagrams (temperature versus time) while the samples were cooling from room temperature to −5 °C or −25 °C. Cooling rates were determined from the slopes of the corresponding curves. In fact, three experiments were performed for each condition and the values of the resulting slopes averaged and taken as the cooling rates to be considered in the subsequent differential scanning calorimetry (DSC) analyses. In this way, cooling rates of 2 °C/min and 6 °C/min were obtained, when solutions were cooled from room temperature to −5 °C and −25 °C, respectively.

In the next step, a differential scanning calorimeter (200 F3, NETZSCH DSC, Selb, Germany) was used to evaluate the crystallization behavior of pure solvent and the polymer solutions. The DSC analyses were carried out by cooling from +40 °C to −50 °C at rates of 2 °C/min and 6 °C/min. The temperature, at which an exothermic peak appeared throughout cooling, was taken into account as the solvent crystallization temperature. The studied samples are summarized in Table 1, with abbreviations according to the HV content and the cooling rates.

### 2.3. SEM and DMTA Analyses of Polymer Scaffolds

A scanning electron microscope (SEM) (VEGA II, TESCAN, Brno, Czech Republic) was used to study the porous structure of PHBV scaffolds. The microstructural features were evaluated from the outer surface and the transverse cross-section of the scaffolds. These cross-sections were obtained by soaking the scaffolds in liquid nitrogen for 2 h before to split them in two parts. Prior to microscopy, the samples were sputter-coated with a thin layer of gold by using a Mitec K950 Sputter Coater (Quorum Technologies Ltd., Ashford, UK).

A dynamic mechanical thermal analyzer (DMTA) (TRITEC DMA 2000, DMA-TRITON, Lincolnshire, UK) was used to estimate the viscoelastic behavior of PHBV scaffolds under dynamic loading conditions (ASTM E1640-04). Polymeric scaffolds (2 cm × 0.7 cm × 0.2 cm) were subjected to cyclic tensile strains of 0.008 mm with frequency of 1 Hz, while temperature was increased from −50 °C to 180 °C at a rate of 5 °C/min. The stress response of samples was recorded via in-phase modulus (*E*′), lag modulus (*E˝*), and loss tangent (*E*˝/*E*′) versus temperature.

### 2.4. Assays of Cell Adhesion and Proliferation

MDCK cells (with epithelial-like morphology and derived from Madin–Darby Canine Kidney, ATCC) and NRK cells (with epithelial-like morphology and derived from the kidney of the *Rattus norvegicus*, ATCC) were employed. Both cell lines grow adherently, and were cultured in Dulbecco’s modified Eagle’s medium (DMEM with 4500 mg/L of glucose, 110 mg/L of sodium pyruvate and 2 mM of l-glutamine) supplemented with 10% fetal bovine serum (FBS), 50 U/mL penicillin, 50 mg/mL streptomycin, and l-glutamine 2 mM at 37 °C in a 10% humidified atmosphere of 5% CO_2_ and 95% air. Culture media were changed every two days. For sub-culture, cell monolayers were rinsed with PBS and detached by incubating them with 0.25% trypsin/EDTA for 2–5 min at 37 °C. The incubation was stopped by resuspending in 5 mL of fresh medium and the cell concentration was determined by counting with Neubauer camera and using 4% trypan blue as dye vital.

HBV5 and HBV12 scaffolds were cut off into pieces of 1 cm × 1 cm. These samples were placed in tissue culture plates of 24-wells and fixed to bottom plate with a small drop of silicone (Silbione^®^ Med Adh 4300 RTV, Bluestar Silicones France SAS, Lyon, France), sterilized by exposed to UV light for 15 min. 100 µL containing 5 × 10^4^ cells/well to assess cell adhesion, and 2 × 10^4^ cells/well for the cell proliferation assay were seeded in each well and incubated for 60 min to allow cell attachment to the material surface. Then, 1 mL of culture medium was added to each well. Quantification of viable cells was performed after 24 h and 7 days to evaluate the cellular adhesion and proliferation, respectively. The control was performed by cell culture on the plate without any material.

The percentage of cells adhered and proliferated was determined through the MTT (3-(4,5-dimethylthiazol-2-yl)-2,5-diphenyltetrazolium bromide) assay [31]. After 24 h or 7 days, 50 µL of MTT (3 mg/mL) were added to each well in the plates and incubated for 4 h. After that, samples were washed twice with PBS and the specimens deposited in a new plate. 1 mL of dimethyl sulfoxide (DMSO) was subsequently added and the absorbance was measured at 570 nm in a microplate reader (Biochrom EZ-Read 400, Cambridge, UK) after 15 min of gentle stirring. Three replicas were evaluated and the corresponding values were averaged and graphically represented. The statistical analysis was performed by one-way ANOVA to compare the average values of all groups; Tukey-test was then applied to determine a statistically significant difference between two studied groups. The tests were performed with a confidence level of 95% (*p* < 0.05).

Samples were fixed overnight with 2.5% formaldehyde in PBS at 4 °C, and then washed five times with PBS to obtain images showing the morphology of cells coming from adhesion and proliferation assays. Samples were also stained to get fluorescence microscopy images. Specifically, actin was labeled with green-fluorescent Alexa Fluor Atto-488 phalloidin dye, and the nucleus was labeled with DAPI (4′,6-diamidino-2-phenylindole). Then, samples were observed using a confocal laser scanning microscope (LSM 900 Zeiss, Oberkochen, Germany), images were taken with a camera controlled by ZEN 2.6 software (blue edition) (Carl-Zeiss Microscopy GmbH, Jena, Germany).

## 3. Results

### 3.1. DSC Testing of Polymer Solutions

**Figure 1:**
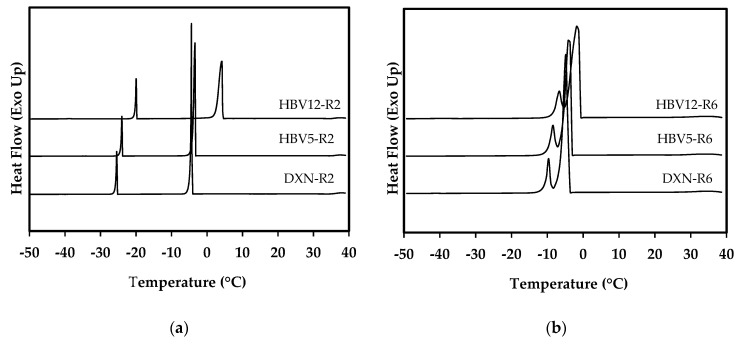
Exothermic peaks observed in the DSC cooling traces of the pure solvent and the two studied copolymer solution samples. Scans were performed at rates of 2 °C/min (**a**) and 6 °C/min (**b**).

DSC results (Figure 1a,b) revealed that crystallization temperature of 1,4-dioxane (*T_c_* of solvent) in the polymer solutions was higher than the pure solvent at the two assayed cooling rates (i.e., 2 °C/min and 6 °C/min).

Additionally, it was observed that the crystallization temperature of 1,4-dioxane was higher at both cooling rates when the copolymer was enriched in HV units (i.e., PHBV(12%HV) solutions gave rise to a higher solvent crystallization temperature than PHBV(5%HV) solutions). DSC cooling runs showed large exothermic peaks associated with the crystallization of 1,4-dioxane, and small peaks related to the well-known reversible phase transition of 1,4-dioxane from its monoclinic phase I to the monoclinic phase II [32].

Neither the large nor the small peaks observed in each cooling trace of the studied polymer solutions could be attributed to a crystallization of the polymer. Note that pure 1,4 dioxane exhibited both mentioned peaks at the same temperature range. According to the values of 1,4 dioxane crystallization temperature in the samples (i.e., −4.3 °C and −4.6 °C for DXN-R2 and DXN-R6; −3.4 °C and −3.9 °C for HBV5-R2 and HBV5-R6; 1.8 °C and −1.5 °C for HBV12-R2 and HBV12-R6), a decrease in *T_c_* of solvent was detected for all the samples without exception, when a higher cooling rate was applied, a feature that was more significant for the copolymer enriched in HV units.

### 3.2. Cloud Point of Polymer Solutions

**Figure 2:**
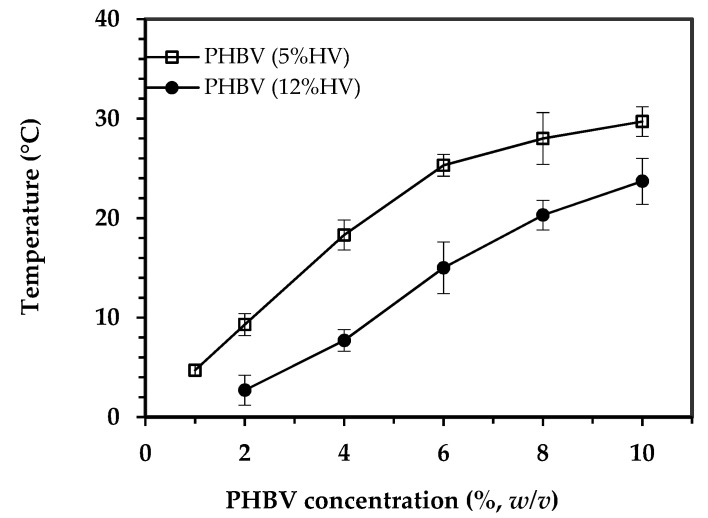
Cloud point curves at relatively low concentrations of PHBV(5%HV)-dioxane and PHBV(12%HV)-dioxane solutions.

Cloud point is a temperature at which a clear polymer solution becomes turbid during cooling because of the liquid–liquid phase separation [26]. The boundary of the liquid–liquid demixing region in the polymer-solvent phase diagram is usually named binodal curve, but the term “cloud point curve” is more appropriate for polydisperse polymers [17]. Figure 2 shows the variation of the cloud point as a function of polymer concentration for the binary systems of PHBV(5%HV)-1,4 dioxane and PHBV(12%HV)-1,4 dioxane.

According to the experimental cloud point curves, a higher cloud point was observed as the polymer concentration increased (at the evaluated concentration range). Additionally, the temperature at which the solution became cloudy decreased with the increase in HV content in the copolymer. Specifically, the cloud point of 2% (*w/v*) solutions of PHBV(5%HV) and PHBV(12%HV), which were used for fabrication of the scaffolds, was 9.3 ± 1.1 °C and 2.7 ± 1.5 °C, respectively. Any trace of cloudy state was not seen in the 1% (*w*/*v*) solution of the PHBV(12%HV) before being frozen. Gelation was also observed to occur before to achieve a cloudy state when concentrated solutions (e.g., higher than 2% (*w*/*v*)) were slowly cooled, especially for the copolymer with lower HV content.

### 3.3. Morphology of Porous Scaffolds

**Figure 3:**
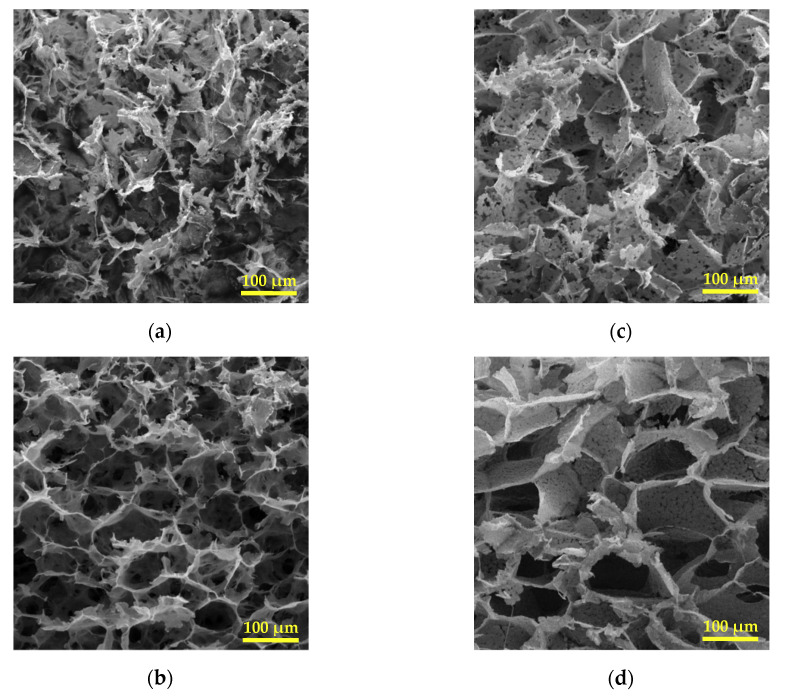
Scanning electron micrographs of cross-sections of PHBV scaffolds: (**a**) HBV5-R2, (**b**) HBV5-R6, (**c**) HBV12-R2, and (**d**) HBV12-R6.

Scanning electron micrographs of split cross-sections (Figure 3) showed that the scaffolds tended to form large pores of around 100 microns with well differentiated walls when underwent phase separation at the higher cooling rate.

**Figure 4:**
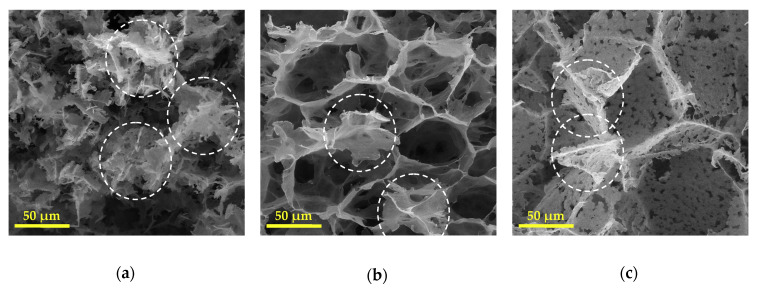
Scanning electron micrographs of cross-sections of PHBV scaffolds: (**a**) HBV5-R2, (**b**) HBV5-R6, (**c**) HBV12-R2. Dashed-line circles present areas detected as platelet-like structures.

It is interesting to note that upon the slower cooling condition, platelet-like structures were mainly distinguished. This structure was also observed to a greater extent in the PHBV(5%HV) copolymer. Some areas representing platelet-like morphology have been indicated by dashed-line circles in Figure 4. Micrographs demonstrated that scaffolds had a three-dimensional porous structure and that the larger pores were further obtained from the scaffolds derived from the copolymer having the higher HV content. Specifically, PHBV(12%HV) scaffolds prepared at the higher cooling rate appear ideal considering the pore sizes, the homogeneous structure and the reduced platelet-like regions.

**Figure 5:**
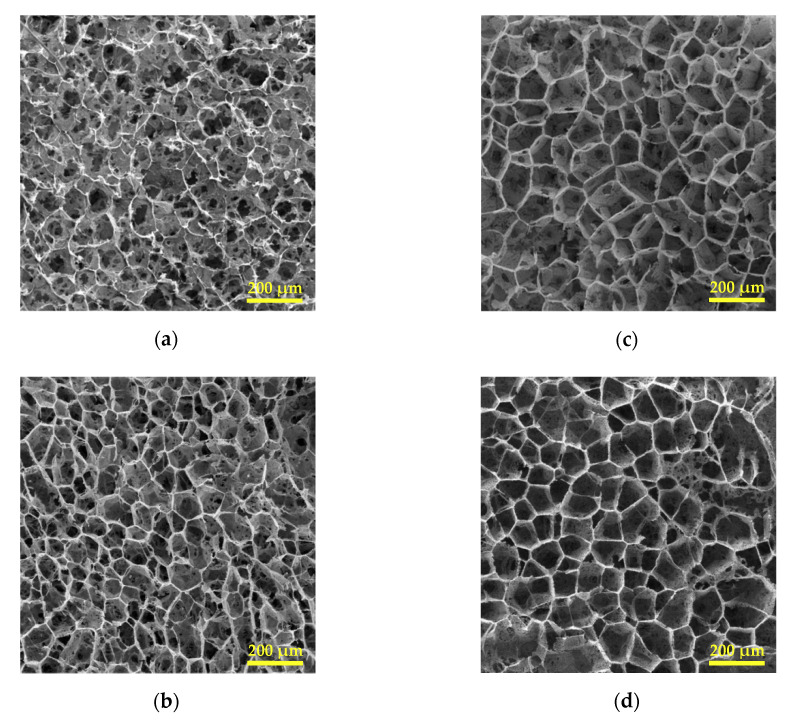
Scanning electron micrographs of the surface of PHBV scaffolds: (**a**) HBV5-R2, (**b**) HBV5-R6, (**c**) HBV12-R2, and (**d**) HBV12-R6.

The tendency towards forming more large pores and reducing platelet-like morphologies were intensified in the surface image micrographs (Figure 5). Images showed again that the HBV12-R6 sample was the more uniform one. The presence of small orifices in the pore walls ranging from several to tens of micron in size were discernible in both cross-section and surface micrographs, being considered as interconnectivities of the structure.

### 3.4. DMTA Analysis of Polymer Scaffolds

**Figure 6:**
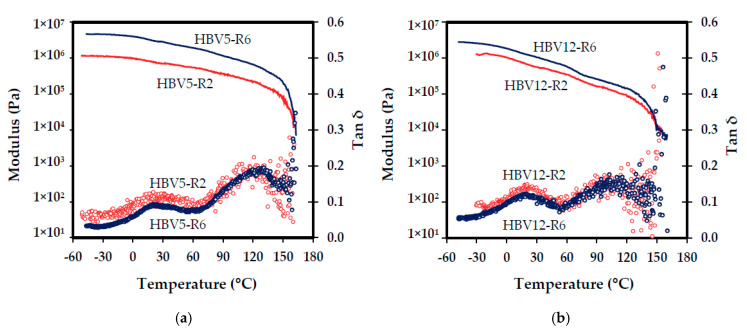
Effect of cooling rate (red and blue symbols/lines correspond to rates of 2 °C/min and 6 °C/min, respectively) on storage modulus (solid-line curves) and loss tangent (circle-marker curves) of PHBV scaffolds containing 5 wt % (**a**) and 12 wt % (**b**) of HV units.

DMTA results revealed that mechanical properties of resulting scaffolds were influenced by the selected cooling rate and logically by the HV molar content of the copolymer, even for the small increase from 5 wt % to 12 wt % (Figure 6).

An increase of the loss modulus (*E*″) and especially of the storage modulus (*E*′) was observed when solutions were cooled at the highest rate. Therefore, the *E*″/*E*′ ratio (i.e., loss tangent or tan δ) decreased. A similar effect was roughly observed when the HV content was lower. In summary, the locus of storage modulus curve shifted to the higher values and that of the loss tangent curve to lower values either by increase in cooling rate or decrease in HV contents. The dramatic decrease in the modulus at temperatures around 160 °C is associated with the melting point of the polymer, in accordance with supplier’s specifications.

### 3.5. Biocompatibility Assays for Scaffolds Prepared by TIPS from 1,4-Dioxane

**Figure 7:**
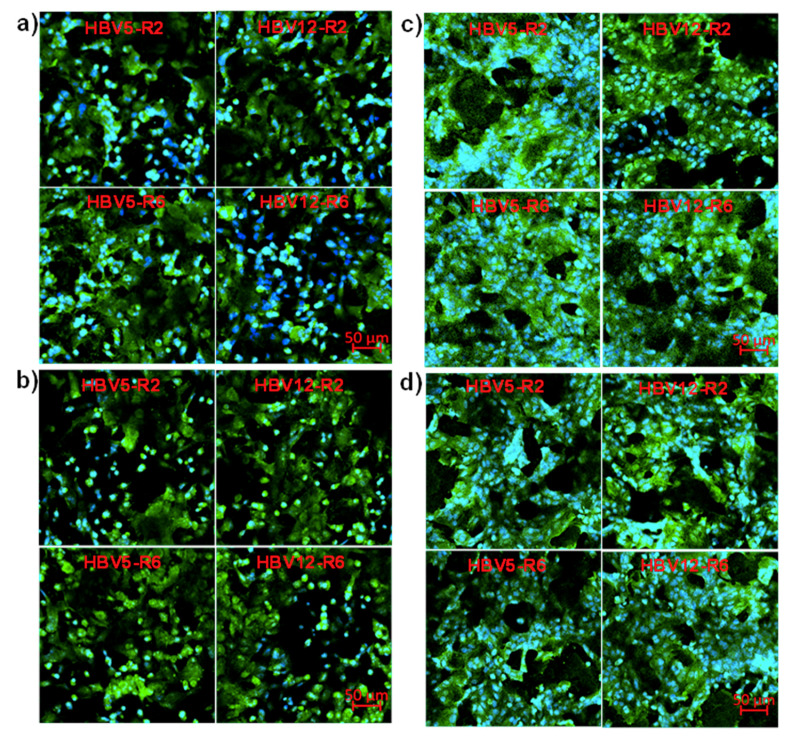
Fluorescence micrographs of NRK (**a**,**c**) and MDCK (**b**,**d**) epithelial cells after performing adhesion (**a**,**b**) and proliferation (**c**,**d**) assays onto HBV5 and HBV12 scaffolds obtained from 1,4-dioxane solutions cooled at rates of 2 and 6 °C/min. The green color is the actin marked with phalloidin and the blue color is the nucleus marked with DAPI.

HBV5 and HBV12 samples were evaluated as appropriate scaffolds to support cell adhesion and proliferation. Thus, epithelial-like MDCK and NRK cells were seeded in direct contact with the prepared scaffolds (Figure 7). Cell adhesion was determined after 24 h as an early event of the cell growth in the scaffolds, while cell proliferation was determined after 7 days to demonstrate that cell growth and colonization were effective in the prepared scaffolds.

Images of fluorescence microscopy gave evidences of the cell adhesion (Figure 7a,b) and of the formation of a cell monolayer onto all the scaffold samples (Figure 7c,d). In the cell adhesion assay, cells appeared spread onto the surface of the scaffolds and the porous structure was maintained as evidenced by the dark and deep zones. In the cell proliferation assay, there was a clear increase of the number of cells grown on the surface of the sample. Micrographs showed that MDCK and NRK cells grew normally to contact each other and formed a cell monolayer by clusters and stackings, being drawn to the profile of the pores in the scaffold. Cells had a smaller size after proliferation due to its density increase, and the porous scaffold structure was maintained as deduced from the dark and deep zones. In this way, the prepared scaffolds had a sufficiently large pore size to not restrict the entry of cells into the scaffolds. The sponge-like morphology of these scaffolds (Figure 5) is compatible with the excellent biocompatibility demonstrated in both cell adhesion and proliferation assays. Results confirmed that the studied scaffolds had a great potential for applications focused on tissue regeneration and remodeling.

**Figure 8:**
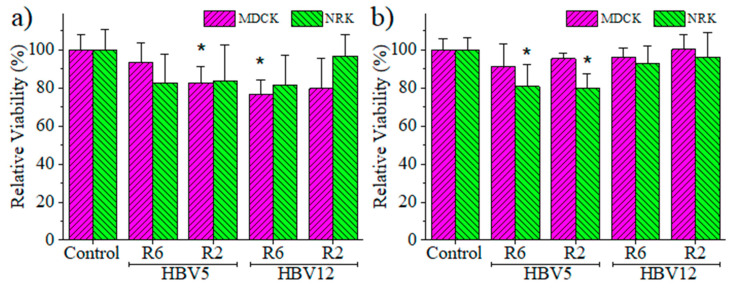
Cell viability of NRK and MDCK epithelial cells for adhesion (**a**) and proliferation (**b**) assays in the HBV5 and HBV12 scaffolds obtained from 1,4-dioxane solutions cooled at rates of 2 and 6 °C/min. * *p* < 0.05 vs. control.

Quantitative data of cell adhesion and proliferation are shown in Figure 8a,b, respectively. The cell viability was determined considering the ratio between the number of cells grown in the scaffold and on the control (well of the culture plate), respectively.

Results indicated that cell adhesion was quantitatively similar in the prepared scaffolds and the control. Only HBV5-R2 and HBV12-R6 samples showed a significant reduction in the number of adhered MDCK cells, with values around 80% of cell viability. However, the samples HBV12-R2 with MDCK cells and HBV5-R6 with NRK cells were not significantly different despite having average values around 80% viability due to the greater dispersion of data as evidenced by their respective standard deviations (Figure 8a). Regarding cell proliferation, which is a more consistent experiment because it corresponds to a period of 7 days, it was observed that the MDCK cells showed similar growth percentages as the control, while the NRK cells in the HBV5 samples showed a significant growth reduction. However, the measured values are close to 80% of viability (Figure 8b), which can cause us to consider that differences may be caused by uncontrolled experimental factors. In this sense, it should be indicated that volume of scaffolds should be taken into account instead of surface (1 cm × 1 cm square samples were analyzed) since the scaffold galleries allow cell entry and colonization inside the scaffold. These considerations are supported by the morphological evidence of fluorescence microscopy for both adhesion and proliferation assays (Figure 7). Therefore, results allow us to indicate that HBV5 and HBV12 scaffolds obtained from the 1,4-dioxane solutions at both 2 and 6 °C/min cooling rates are suitable and biocompatible supports for cell adhesion and proliferation in 3D cultures, and show potential interest for tissue regeneration applications.

## 4. Discussion

### 4.1. Solvent Crystallization Temperature

Since TIPS is a non-equilibrium process, the effects of the cooling rate on the phase diagram must be regarded [33]. Based on DSC results, higher cooling rates led to a slight reduction of solvent *T_c_* in all samples. Increasing the cooling rate allows getting a higher supercooling. Namely, the solution can be cooled to a temperature below its equilibrium crystallization temperature, avoiding crystallization of the solvent from the solution [34].

As it was reported before, the crystallization temperature of 1,4-dioxane in the polymer solutions was higher than pure 1,4-dioxane, meaning that the presence of PHBV has provoked the earlier crystallization of 1,4-dioxane during cooling. Crystallization of 1,4-dioxane took place according to a typical mechanism consisting on nucleation and growth steps [23,35]. It could be assumed that crystallization temperature of the solvent becomes higher (anticipation of crystallization during cooling) when the nucleation process is facilitated. Therefore, the incorporation of poly(3-hydroxybutyrate-*co*-3-hydroxyvalerate) in 1,4-dioxane may favor the formation of nuclei. Note that there are hydroxyl and carboxyl functional groups on both ends of a PHBV chain and two exposed oxygen atoms in the molecular structure of 1,4 dioxane. Interactions between these functional groups and the oxygen lone pair electrons of dioxane are not negligible [36]. In fact, it should be expected that hydrogen bonds were established between 1,4-dioxane and the molecular chains [37]. In this way, the hydrogen bonded molecules of solvent and polymer should have a restricted movement and displacement at the site of binding [38]. These solvent molecules with finite mobility might serve as preferential nucleation sites and facilitate the solvent crystallization.

PHBV(12%HV)-dioxane solutions showed higher *T_c_* of solvent than PHBV(5%HV)-dioxane solutions. Two points may justify this observation: Its lower molecular weight and its lower crystallinity. On one hand, it seems that the increase of functional groups (i.e., terminal groups) should increase the interactions with the solvent. On the other hand, diffusion of the solvent molecules should be easier when the copolymer becomes less crystalline. Therefore, the capability to form hydrogen bonding interactions becomes increased due to the higher accessibility of hydroxyl and carboxyl groups to the solvent molecules. This crystallization behavior is significantly enhanced upon slow cooling, where enough time is available for the molecules to trigger proper interaction sites during the crystallization process.

### 4.2. Cloud Point of Polymer Solutions

At experimentally controllable cooling rates, a minor effect of the cooling rate on the liquid–liquid phase separation temperature and the location of the cloud point curve has been reported [39]. Therefore, only a slow cooling (i.e., a constant rate of 1 °C per 30 min) was applied to reach the equilibrium conditions for the cloud point determination [26]. Logically, the cloud point temperature decreased as the HV content in the copolymer increased.

**Figure 9:**
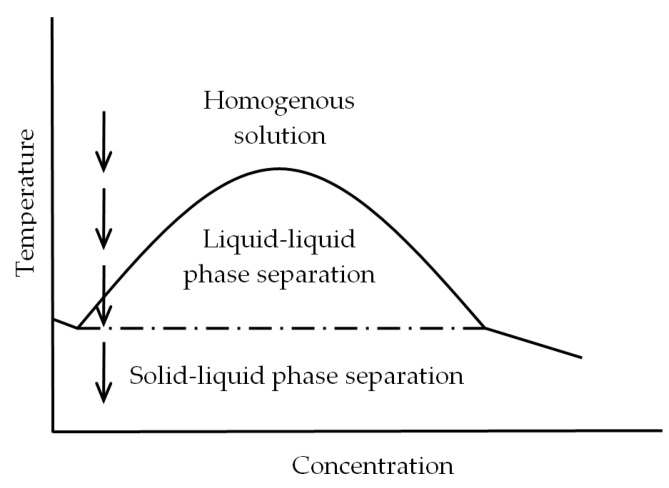
Typical phase diagram for a polymer-solvent system showing the regions of liquid–liquid and solid–liquid phase separation. Arrows schematically indicate the phase separation evolution during cooling for an initial solution with a low (i.e., close to 2%) polymer concentration.

The Flory–Huggins equation for the polymer-solvent system is [40]

(1) $$\frac{\Delta G_{mix}}{RT}=\frac{\phi_{d}}{x_{d}}ln\phi_{d}+\frac{\phi_{p}}{x_{p}}ln\phi_{p}+\chi \phi_{d}\phi_{p}$$

where *ΔG_mix_* is the Gibbs free energy of mixing per lattice site, *ϕ_d_* and *ϕ_p_* are the volume fraction of solvent and polymer, *x_d_* and *x_p_* are the number of lattice sites occupied by solvent and polymer molecules, respectively. *χ* is the Flory-Huggins interaction parameter which is affected by the strength of polymer–solvent interactions. The first two terms on the right side of the Flory–Huggins equation are always negative and represent combinatorial entropy contribution and the third term can be positive or negative (depending on the sign of *χ*) and represents the enthalpic contribution. Weak polymer–solvent interactions give rise to a large and positive value of *χ*, leading to a positive *ΔG_mix_* and consequently liquid–liquid demixing happens. On condition that the strength of interactions between polymer and solvent is high (small *χ*), it is more difficult to attain the liquid–liquid phase separation conditions (i.e., a lower temperature is needed). In this situation, the homogenous one-phase region in the phase diagram (Figure 9) expands, and so the boundary of liquid–liquid phase separation (binodal curve) shifts to lower temperatures [33]. Accordingly, the lower cloud point of PHBV(12%HV) solution is speculated to be the result of its stronger interactions with 1,4-dioxane, compared to the other copolymer. As it was elaborated in the previous section, the lower molecular weight and crystallinity of PHBV(12%HV) results in an increased number of interactions with 1,4-dioxane.

### 4.3. Phase Separation Mechanisms

TIPS can be divided into two major types of separation processes: liquid–liquid and solid–liquid phase separations. The former may occur prior to the solvent freezing, and the latter is only observed when the solvent has been completely frozen [41]. In the case where the solvent crystallization temperature in the solution is higher than the liquid–liquid phase separation temperature, the solid–liquid phase separation occurs during the cooling process [21]. After removing the solvent, the polymeric foam is characterized by pores having a similar geometry of solvent crystallites [17,23,25], namely a pore size of around 100 microns remains [41]. The liquid–liquid phase separation takes place, if the solvent crystallization temperature is much lower than the phase separation temperature. Morphologically, this type of phase separation produces a continuous isotropic structure with pores ranging from several to tens of microns. On condition that the solution involves a semicrystalline polymer, it will encounter driving forces for both liquid–liquid phase separation and polymer crystallization, due to the crystallization potential of the polymer. A crystallization-induced phase separation (another type of solid–liquid phase separation) can occur in this situation, if the polymer crystallization temperature is higher than the phase separation temperature and the solution is held long enough at a temperature above the phase separation temperature [21]. Depending on the polymer concentration, the crystallization or precipitation of polymer from the solution can lead to different morphologies varying from loose precipitates (i.e., unconnected precipitates) to percolating structures (i.e., interconnected networks of crystallites) [17]. In this regard, various microstructures such as platelet-like structures from relatively low concentrations [27] and spherulitic structures from relatively concentrated solutions have been reported [33,34].

A typical phase diagram for the polymer–solvent binary system, corresponding to thermodynamically favored phase transitions of this study is depicted in Figure 9. The concentration used for fabricating the polymeric foams is schematically showed by a sequence of arrows orientated towards lower temperatures. Upon cooling, the solution crosses the binodal curve (solid curve), undergoing liquid–liquid phase separation by nucleation and growth or spinodal decomposition mechanism. As cooling continues and the temperature falls below the solvent crystallization temperature (dashed horizontal line), the solution subsequently experiences the solid–liquid phase separation [21,26]. Regarding the roughly adjacent temperatures measured for the cloud point and solvent crystallization in the solutions, a limited period of time is expected between these two-phase separation types when cooling. Depending on cooling rates and HV contents, the solutions underwent solid–liquid phase separation immediately or in slightly delayed manner after the liquid–liquid demixing, resulting in relatively different morphologies and mechanical properties. Materials with relatively different morphologies and mechanical properties are consequently derived according to the phase separation process.

Considering the PHBV melting temperature of about 160 °C, it can be deduced that PHBV is crystallized from the solution at temperatures higher than the range that we studied in the DSC experiments. Due to existence of a driving force for the crystallization of the polymer at relatively elevated temperatures, this phenomenon is thermodynamically favored until the binodal curve is reached by the solution throughout cooling. Nevertheless, the crystallization-induced phase separation is significantly dependent on the kinetic conditions that have been elaborated in the next section. The gelation observed via slow cooling at relatively high temperatures (above the liquid–liquid phase separation temperature—cloud point) can be attributed to polymer crystallization [27].

Although it is not possible to determine the exact contribution of these three phase separation types in this study, the qualitative changes in their corresponding microstructures (caused by changing the cooling rate and HV contents) is discernible in the SEM micrographs (Figure 3 and Figure 5).

### 4.4. Phase Separation and Morphology Relationship

Scaffold morphologies obtained at different cooling rates can be well explained by analyzing the phase separation process. It should be noted that an increase in the cooling rate results in a rapid temperature decrease from that associated with the binodal curve to that corresponding to the solvent crystallization horizontal line. In other words, the time at which the system remains in the liquid–liquid separation region becomes minimum (Figure 9). On the other hand, higher cooling rate led to a slight decrease in the solvent crystallization temperature and therefore causes an insignificant enlargement of the indicated temperature interval (binodal-solvent crystallization).

Large pores of around 100 microns are usually observed at higher cooling rate in contrast with the great number of small pores (i.e., several microns) that are observed at lower cooling rate. As a consequence, it can be morphologically inferred that crystallization of the solvent bypass the liquid–liquid phase separation under the higher cooling rate conditions used in this study. Similarly, Zhang and Ma showed that when polymer solutions (with several different solvent systems) were cooled fast enough and to a temperature low enough, the solid–liquid phase separation occurred, because there was not enough time for the liquid–liquid phase separation to take place [23].

The prominent effect of the cooling rate on the morphology of the studied system corresponds to the reduction of platelet-like morphologies ascribed to the polymer crystallization. Despite the fact that, above the binodal curve, polymer crystallization is thermodynamically favored during cooling, the kinetics of phase separation specifies that whether the thermodynamically favored transition happens or not, and also to what extent the transition occurs [17].

Since nucleation and growth of polymer crystals from the solution is a slow process [17], it may not happen or happen deficiently upon rapid cooling. In this regard, it has been reported that high cooling rates avoid the nucleation and growth of polymer crystals, being also indicated that platelet-like structures increased with the annealing time at room temperature [27].

The both mentioned morphological effects caused by increase in the cooling rate, especially the reduction of platelet-like zones were clearly observed by comparison of cross-section images (Figure 3) and surface images (Figure 5) for each sample. The observed differences may be due to an unwanted temperature gradient from the surface to the center of the samples during cooling, resulting in different local cooling rates.

From the copolymer type viewpoint, in PHBV(12%HV), the distance between binodal curve and solvent crystallization line decreased in three ways. Firstly, the locus of binodal curve and the intersection point of binodal curve and solvent crystallization line shifted to the right. As it was previously described, the 1% (*w*/*v*) solution of PHBV(12%HV) unlike the same concentrated solution of PHBV(5%HV), was frozen before being cloudy, meaning that the former sample has only crossed the solvent crystallization line at the left side of the intersection point. Secondly, solvent crystallization temperature in the solutions became higher. Thirdly, the locus of binodal curve shifted to lower temperatures. Getting smaller the mentioned temperature interval in the phase diagram, the liquid–liquid demixing and the solvent crystallization almost occur simultaneously in PHBV(12%HV)-dioxane solution. This situation results in leaving little room for growth of the polymer-lean droplets formed by liquid–liquid phase separation. Hence, most of the pores arising from these droplets might not grow properly to reach tens of microns in size; instead, they remain in the form of small orifices of several microns trapped throughout walls of larger pores (Figure 3c,d). Additionally, the solutions roughly tended to form structures dominated by large pores of around 100 microns pertaining to solid–liquid phase separation (particularly at higher cooling rates).

Regarding the obtained morphologies of split cross-sections (Figure 3), it can be concluded that the highest contribution of solid–liquid phase separation and therefore the highest number of large pores are related to sample HBV12-R6, and the lowest one to sample HBV5-R2. The latter sample also possessed the highest contribution of crystallization-induced phase separation and its corresponding morphology. The sample HBV12-R2 exhibited a platelet-like structure along with the small pores of around 5 microns. Furthermore, the sample HBV5-R6 obviously shows a variety of structures comprising the large pores, the small pores which have grown up to 10–20 microns and also the platelet-like regions, ascribed to three mentioned types of phase separation. Conclusively, crystallization of the polymer seems to be the premier mechanism under slower cooling conditions, while the crystallization of the solvent was the prominent mechanism for samples having the highest HV content.

### 4.5. Structural Characteristics and DMTA Results

According to DMTA results, increase in cooling rate and decrease in HV content of PHBV samples resulted in higher storage modulus and lower loss tangent, which are indicative of scaffold rigidity and damping, respectively (Figure 6). Strictly speaking, viscoelastic behavior of scaffolds is characterized by a higher strength and a lower viscose contribution under the higher cooling rate and lower HV content conditions. The better mechanical properties of the copolymer with lower HV content could be further attributed to its higher crystallinity.

On the other hand, upon slower cooling, the regions having morphologies arising from the polymer crystallization increased. These regions are made up of platelets of polymer rich phase (formed by nucleation and growth of polymer crystals through slow cooling) suspended in a matrix of polymer-lean phase [21]. The regions, which are formed by crystallization of the polymer from dilute solutions, have been characterized by insufficient structural consistency and mechanical properties [27,34]. For example, when PLLA crystallized slow enough from a 5 wt % solution, a loose connection between the platelets was reported by Ma and Zhang [27]. Therefore, weak mechanical properties were justified for the platelet-like structured matrices. Lloyd et al. also produced leafy architectures with structural consistency, when HDPE crystallized from 15–50 wt % solutions, while no structural integrity was reported at concentrations below 15 wt % [34]. Regarding the concentration of the solutions used for fabricating the scaffolds in this study (2% *w*/*v*), a poor structural connection and therefore a mechanical weakness should be expected for the platelet-like regions. Since the amount of these regions is increased by slower cooling, a decrease in the mechanical performance is expected at the lower cooling rate condition as it is confirmed by DMTA results.

On the other hand, the lower HV molar ratio raised crystallinity of PHBV, and so, enhanced mechanical properties can be attributed to this reality. The increase in the storage modulus caused by the decrease in the HV contents is clearly more significant in the rubbery region with respect to the glassy region. It can be postulated that the increase in density and crystallinity led to a high restriction of the segmental motion of the polymer chains in the rubbery region, while a lower influence was derived in the glassy region characterized by limited molecular vibrations.

### 4.6. Biocompatibility of Porous PHBV Scaffolds

Current tissue engineering strategies focus on the reconstruction and regeneration of damaged tissues. The use of porous scaffold biomaterials becomes an interesting issue in reparative medicine. To restore the functionality of a tissue, the presence of a biodegradable scaffold can be essential as an extracellular matrix for cell colonization, migration, growth, and differentiation, until the tissues are restored or regenerated completely. Great attention has been paid to the PHBV bioplastic due to its potential biomedical applications. In this sense, our current work with porous PHBV substrates could be pioneering in demonstrating the good capacity of these substrates to promote adhesion and proliferation of epithelial cells. Our results showed that porous PHBV scaffolds allow the adhesion of a high percentage of epithelial cells (e.g., equal to or greater than 80%). However, our proliferation results at 7 days also indicated that HBV5 scaffolds supported a scarcely lower proliferation of NRK cells, a feature that could be related to the high crystallinity and stiffness of this material. Fortunately, MDCK cells showed an excellent growth on these porous matrices. In vitro tests appear therefore indicative but not conclusive, being required evidence from in vivo tests to improve conclusions about biocompatibility. The results obtained in this work are sustained for a wide literature data about the excellent biocompatibility of PHBV. Several techniques for the PHBV scaffold fabrication have been developed in the last decades: polymerization in solution, leaching, electrospinning, and 3D printing [42]. PHBV matrices have been developed for both hard [43] and soft [44] tissue engineering applications, e.g., bone and skin, respectively. In particular, epithelial-like cells such as UMR-106 osteoblast maintain their phenotypic characteristics in PHBV matrices [45]. Furthermore, in similar applications for skin engineering, the fiber matrices based on PHBV and obtained by electrospinning demonstrated that human skin fibroblasts (CRL 2072) were able to adhere and colonize these new substrates [44]. Different PHB organogels and scaffolds with complex hierarchical structure and covering a wide range of length scales have been prepared by TIPS and showed an excellent cell viability using the human keratinocyte cell line (HaCaT) [46]. Finally, our results contribute to the suggestion of the great application of PHBV in tissue engineering.

## 5. Conclusions

The principal aim of this work was the study of process-properties relationship for poly(hydroxybutyrate-*co*-hydroxyvalerate) scaffolds fabricated by thermally induced phase separation. Due to presence of different driving forces for polymer and solvent crystallization and also liquid–liquid demixing, the phase separation process became relatively complicated. Thus, it was hard to consider a distinctive mechanism, being responsible for generating the different microporous structures. Variables corresponding to the fabrication process (i.e., cooling rate applied to the polymer solution) and material selection (i.e., PHBV with different HV molar ratios) strongly affected the phase separation process and led to different microporous structures and mechanical properties of the resulted scaffolds. Strictly speaking, more regions having the morphology associated with crystallization of the polymer were conspicuously detected upon slower cooling. These regions of relatively poor structural continuity were assumed to be the reason of the observed decrease in rigidity of the scaffolds. Besides, a tendency towards forming the typical morphology related to a solid–liquid phase separation was also observed in either higher cooling rate or higher HV content. Eventually, a high degree of crystallinity was recognized as the cause for the higher rigidity of the scaffolds having lower HV content. Finally, in-vitro cytocompatibility studies confirmed that these sponges-like scaffolds were nontoxic toward MDCK and NRK cells, and had a suitable porosity to cell adhesion and growth. Our data demonstrated that these biocompatible scaffolds with interconnected 3D networks are a promising to applications focused on tissue regeneration and remodeling.

## Figures and Tables

**Table 1:** Sample designation

Sample	Cooling Rate (°C/min)	Abbreviation
Pure dioxane	2	DXN-R2
Pure dioxane	6	DXN-R6
PHBV(5%HV)-dioxane	2	HBV5-R2
PHBV(5%HV)-dioxane	6	HBV5-R6
PHBV(12%HV)-dioxane	2	HBV12-R2
PHBV(12%HV)-dioxane	6	HBV12-R6
